# Melatonin receptor 1 B polymorphisms associated with the risk of gestational diabetes mellitus

**DOI:** 10.1186/1471-2350-12-82

**Published:** 2011-06-10

**Authors:** Jason Y Kim, Hyun Sub Cheong, Byung-Lae Park, Sei Hyun Baik, Sunmin Park, Si Won Lee, Min-Hyoung Kim, Jin Hoon Chung, June Seek Choi, Moon-Young Kim, Jae-Hyug Yang, Dong-Hee Cho, Hyoung Doo Shin, Sung-Hoon Kim

**Affiliations:** 1 Department of Life Science, Sogang University, 1 Shinsu-dong, Mapo-gu, Seoul, 121-742, Republic of Korea; 2 Department of Genetic Epidemiology, SNP Genetics, Inc.,1407 14th Floor, Woolim lion's valley B, Gasan-dong, Geumcheon-Gu, Seoul, 153-803, Republic of Korea; 3 Department of Internal Medicine, Korea University College of Medicine, Seoul, Republic of Korea; 4 Department of Food & Nutrition, Hoseo University, Asan-Si, Republic of Korea; 5 Department of Obstetrics and Gynecology, Cheil General Hospital and Women's Healthcare Center, Kwandong University College of Medicine, Seoul 100-380, Republic of Korea; 6 Department of Laboratory Medicine, Cheil General Hospital and Women's Healthcare Center, Kwandong University College of Medicine, Seoul 100-380, Republic of Korea; 7 Department of Medicine (S.-H.K.), Cheil General Hospital and Women's Healthcare Center, Kwandong University College of Medicine, Seoul 100-380, Republic of Korea

## Abstract

**Backgrounds:**

Two SNPs in *melatonin receptor 1B *gene, *rs10830963 *and *rs1387153 *showed significant associations with fasting plasma glucose levels and the risk of Type 2 Diabetes Mellitus (T2DM) in previous studies. Since T2DM and gestational diabetes mellitus (GDM) share similar characteristics, we suspected that the two genetic polymorphisms in *MTNR1B *may be associated with GDM, and conducted association studies between the polymorphisms and the disease. Furthermore, we also examined genetic effects of the two polymorphisms with various diabetes-related phenotypes.

**Methods:**

A total of 1,918 subjects (928 GDM patients and 990 controls) were used for the study. Two *MTNR1B *polymorphisms were genotyped using TaqMan assay. The allele distributions of SNPs were evaluated by *x*^2 ^models calculating odds ratios (ORs), 95% confidence intervals (CIs), and corresponding *P *values. Multiple regressions were used for association analyses of GDM-related traits. Finally, conditional analyses were also performed.

**Results:**

We found significant associations between the two genetic variants and GDM, *rs10830963*, with a corrected *P *value of 0.0001, and *rs1387153*, with the corrected *P *value of 0.0008. In addition, we also found that the two SNPs were associated with various phenotypes such as homeostasis model assessment of beta-cell function and fasting glucose levels. Further conditional analyses results suggested that *rs10830963 *might be more likely functional in case/control analysis, although not clear in GDM-related phenotype analyses.

**Conclusion:**

There have been studies that found associations between genetic variants of other genes and GDM, this is the first study that found significant associations between SNPs of *MTNR1B *and GDM. The genetic effects of two SNPs identified in this study would be helpful in understanding the insight of GDM and other diabetes-related disorders.

## Background

The prevalence of type 2 diabetes mellitus (T2DM) in Korean population has dramatically increased over last decades. Although Asian populations traditionally had a low percentage of T2DM patients, it has increased drastically in recent decades. This is largely due to the fact that Asian countries are adopting western lifestyle and diets. However, a recent discovery of diabetes-susceptible loci on human chromosomes suggest that genetic factors may also play a role in the disease development [1].

Gestational diabetes mellitus (GDM) is a condition in which pregnant women exhibit glucose intolerance in various degrees [2], affecting approximately 2-14% of pregnancies [1,3,4]. Women with GDM show similar physiological and genetic characteristics found in diabetes outside of pregnancy, and not surprisingly, women with GDM possess higher risk for developing T2DM when they are not pregnant. Therefore, studying GDM is a good way to study early pathogenesis of diabetes and possibly develop treatment or remedy for the disease [5]. However, while genetic studies on T2DM are very robust [6,7], there are relatively fewer genetic studies for GDM.

Advance of technology in the genetics field has provided us with a number of useful tools to study human genome. Among them, genome wide association studies (GWAS) are a powerful and useful way to detect genes associated with various diseases, including diabetes. Recently, a couple of studies have revealed that the genetic variants in *melatonin receptor 1 B *(*MTNR1B*) gene are associated with T2DM and fasting glucose levels [8,9]. *MTNR1B *gene encodes MT2 protein which, along with MT1 protein encoded by *MTNR1A*, is one of two high-affinity forms of melatonin receptor. This gene product is also an integral membrane protein forming a G-protein coupled 7-transmembrane receptor. Melatonin, also known chemically as N-acetyl-5-methoxytryptamine, is a primary neurohormone secreted by pineal gland. It is mostly found in retina and brain, and its main function is thought to be the regulation of circadian rhythm by translating photoperiodic information from the eyes to the brain. There have been some studies suggesting that insulin level is regulated by circadian clock. Furthermore, T2DM patients have exhibited impaired melatonin secretion and circadian rhythm [10].

To date, several studies have shown the association between *MTNR1B *and T2DM [6,11], but there has yet to be a study which looked into the association between *MTNR1B *and GDM. Since GDM shares many clinical features with T2DM, there is a high possibility that *MTNR1B *is associated with predisposition of GDM. Therefore, we conducted an association study between two polymorphisms of *MTNR1B*, which were previously associated with T2DM, using 928 GDM patients and 990 controls. In addition, we also examined the possible association between the two SNPs of *MTNR1B *and clinical phenotypes related to GDM, such as insulin sensitivity and beta-cell function.

## Methods

### Subjects

All 1,918 subjects included in this study were of Korean ethnic origin and recruited from Cheil General Hospital in Seoul, Korea, from 2003 to 2009. The clinical profiles of the patients are summarized in Table 1. All pregnant women who had not been previously diagnosed with T2DM were screened for GDM using a universal two-step GDM screening program at 24-28 week during gestation (GDM patients mean gestational week = 26.03 ± 2.69, NGT (Normal glucose tolerance) patients mean gestational week = 26.12 ± 1.69, Table 1). The first step was a 50-g glucose challenge test; if the result was positive (plasma glucose levels over 7.8 mmol/liter) after 1 hour, it was followed by conducting a 100-g, 3-hour oral glucose tolerance test (OGTT) after overnight fast according to criteria outlined by Carpenter and Coustan [12]. The threshold glucose values were as follows: fasting, at least 5.3 mmol/liter, 1 hour, at least 10.0 mmol/liter, 2 hours, at least 8.6 mmol/liter, and 3 hours, at least 7.8 mmol/liter. Plasma glucose concentrations were measured by the glucose oxidase method using a YSI 2300 STAT (YSI; Yellow Springs, Ohio). Insulin concentrations were measured using a human-specific radioimmunoassay kit (Linco Research, St. Charles, MO). If two or more of the glucose values were met or exceeded the above thresholds, GDM was diagnosed. A total of 928 GDM women were included in this study. 299 who were negative for 50-gr glucose challenge test and 691 who were pregnant and identified as normal glucose tolerant after undergoing the 100 gr. Glucose challenge test. The homeostasis model assessment (HOMA) index was used to calculate beta-cell function (HOMA-B) and insulin resistance (HOMA-IR) in various populations [13,14]. Our study conforms to the Helsinki declaration and also to the Korean legislation. The institutional review board of Cheil General Hospital approved the study, and all subjects in the study provided informed consent.

**Table 1:** Clinical profiles of subjects

Profiles	GDM	Controls	*P *value
Number of subjects	928	990	
Age(yr)	33.17 (22-52)	32.24 (23-44)	<0.0001
Mean gestation week (wk)	26.03 ± 2.69	26.12 ± 1.69	0.37
BMI(kg/m2)	23.32 ± 4.01	21.40 ± 2.93	<0.0001
AUC-G (Area under glucose curve)	482.46 ± 57.04	358.72 ± 39.99	<0.0001
HOMA-B (Homeostatic model assessment, β-cell function)	208.06 ± 112.75	268.30 ± 179.88	<0.0001
HOMA-IR (Homeostatic model assessment, insulin resistance)	3.07 ± 1.76	2.14 ± 1.02	<0.0001
Fasting plasma insulin (pmol/liter)	13.51 ± 6.62	10.82 ± 4.72	<0.0001
Fasting plasma glucose (pmol/liter)	89.95 ± 13.72	79.31 ± 6.11	<0.0001

Data are presented as means ± standard deviation except age. Data are presented as SD.

### SNP genotyping

Two polymorphisms of *MTNR1B *previously reported in a T2DM association study were selected and were genotyped using a TaqMan [15] assay in the Korean population. Genotyping quality control was performed in 10% of the samples by duplicate checking (rate of concordance in duplicates > 99%). The genotyping call success rates were 98.07% and 97.71% for *rs1387153 *and *rs10830963*, respectively. The probes used were C_1932612_10 for rs1387153 and C_3256858_10 for rs10830963.

### Statistical analyses

The allele distributions of polymorphisms among GDM patients and normal subjects were evaluated by *x*^2 ^models calculating odds ratios (ORs), 95% confidence intervals (CIs), and corresponding *P *values. We used SAS version 9.1 (SAS Inc., Cary, NC) for the calculation. Multiple regressions were used for association analyses of GDM-related phenotypes adjusting for age and body mass index (BMI) as covariates, also using SAS version 9.1. Linkage disequilibrium between the two SNPs were calculated by the Haploview v4.1 software downloaded from the Broad Institute http://www.broadinstitute.org/mpg/haploview[16]. Statistical power of association was calculated by using Power for Genetic Association software [17]. For the calculation, disease prevalence of GDM was estimated to be 3%, based on previous researches [18], with risk allele frequencies of 0.503 and 0.521 for *rs1387153 *and *rs10830963 *respectively, and odd ratios of 1.3 and 1.35, also for respective polymorphisms. With these parameters, it was calculated that our sample of 928 cases and 990 controls would have over 90% statistical power with a type I error rate of 0.05. In order to correct the data for multiple testing, Bonferroni correction was applied. Also, we used PHASE software for haplotype inference [19], and inferred haplotypes were analyzed using SAS version 9.1 for the logistic analyses.

Also, we used PHASE software to estimate individual haplotypes and their frequencies, which uses a Bayesian approach. Individuals with phase probabilities less than 97% were excluded in analysis. To analyze the associations of haplotypes, we used Haplo.stats http://mayoresearch.mayo.edu/schaid_lab/software.cfm, which provides several haplotype-specific tests for association, as well as adjustment for non-genetic covariates and computation of simulation *P*-values. We also conducted conditional analyses with PLINK software http://pngu.mgh.harvard.edu/~purcell/plink/.

## Results

Nine hundred and twenty eight GDM patients were recruited for the present study, and we also recruited 990 pregnant women with normal glucose tolerance as controls. The clinical profiles of the subjects are summarized in Table 1, with characteristics related with T2DM such as the area under glucose curve (AUC-G), fasting plasma insulin (FPI), and fasting plasma glucose (FPG). We also obtained homeostatic model assessment data for both groups in beta-cell function and insulin resistance (HOMA-B and HOMA-IR, respectively). Most of the phenotypes investigated for the subjects showed significant difference between the GDM patients group and the control group, (Table 1, P value < 0.0001 for all phenotypes except mean gestational week), which was to be expected because the phenotypes that showed the significant differences were associated with the diabetic condition. GDM patients were older, possessed higher BMI than the NGT women and clearly exhibited the clinical characteristics of T2DM, as shown in Table 1; in comparison to NGT women, GDM patients exhibited higher blood glucose levels and lower beta-cell function when insulin resistance was increased.

We first performed association analyses of the two genetic polymorphisms in GDM and non-GDM subjects to determine whether these polymorphisms were associated with a higher risk of developing GDM, which were previously found to be associated with T2DM (*rs1387153 *and *rs10830963 *on *MTNR1B)*, with the risk of GDM (Table 2). The risk allele frequencies of both SNPS are also shown in Table 2. As a result, both SNPs showed significant associations with GDM in co-dominant, dominant, and recessive models. Co-dominant model exhibited lowest *P *value (0.00008, *Pcor*. = 0.0006, OR (95% CI) = 1.30 (1.06 - 1.66) and 0.00001, *Pcor*. = 0.00008, OR = 1.35 (1.18 - 1.54) for *rs1387153 *and *rs10830963*, respectively), and recessive model showed highest *P *value (0.002, OR = 1.42 (1.14 - 1.78) and 0.0001, OR = 1.54 (1.23 - 1.92) respectively for *rs1387153 *and *rs10830963*, respectively), while dominant model *P *value was in between the two (0.0007, OR (95% CI) = 1.44 (1.17 - 1.78) and 0.0006, OR = 1.46 (1.18 - 1.81) for *rs1387153 *and *rs10830963*, respectively). The risk allele for *rs1387153 *was T, and for *rs10830963*, it was G. P-value for Hardy-Weinberg equilibrium of subjects and controls were over 0.05 for both SNPs, indicating that the sample population is in Hardy-Weinberg equilibrium (data not shown). Also, there was a significantly high value of linkage disequilibrium (LD) between the two SNPs (|*D'*| = 0.89). The association results of haplotypes with the 2 SNPs are shown in Table 3 and 4.

**Table 2:** Allele and genotype distributions of *MTNR1B *polymorphisms in GDM and control subjects

Loci	Genotype	RAF		N (%)		Referent		Co-dominant		Dominant		Recessive	
		
		GDM	Controls	GDM	Controls	OR(95%CI)	***Pcor***.	OR(95%CI)	***Pcor***.	OR(95%CI)	***Pcor***.	OR(95%CI)	***Pcor***.
	CC			235(25.9%)	313(32.2%)	1							
	CT			433(47.6%)	455(46.8%)	1.33	0.1	1.3	**0.0008 **	1.44	**0.008 **	1.42	**0.03 **
*rs1387153*		0.503	0.444			(1.06-1.66)		(1.14-1.49)		(1.17-1.78)		(1.14-1.78)	
	TT			241(26.5%)	204(21.0%)	1.29	**0.001**						
						(1.13-1.47)							

	CC			217(23.9%)	294(30.4%)	1							
	CG			435(47.9%)	469(48.6%)	1.31	0.2	1.35	**0.0001**	1.46	**0.006 **	1.54	**0.001**
*rs10830963*		0.521	0.453			(1.04-1.65)		(1.18-1.54)		(1.18-1.81)		(1.23-1.92)	
	GG			256(28.2%)	203(21.0%)	1.34	**0.0001**						
						(1.18-1.53)							

*P *values were corrected for multiple testing by multiplying the number of tests (10 tests, 2 SNPs × 5 tests (2 referent, co-dominant, dominant and recessive models)) (Bonferroni correction). RAF, risk allele frequency.

**Table 3:** Haplotype association analyses with GDM and GDM-related phenotypes

Haplotype	Frequency	Phenotype						
		
		GDM(case/control)	BMI	AUC_G*	HOMA_B*	HOMA_IR*	FPI*	FPG
*ht1 *(C-C)	0.452	**0.0001**	0.93	**0.0004**	**0.002**	0.19	0.35	**0.01**
*ht2 *(T-G)	0.486	**0.000001**	0.8	**0.00007**	**0.002**	0.08	0.23	**0.002**
*ht3 *(C-G)	0.045	0.25	0.15	0.25	0.8	0.66	0.94	0.17
*ht4 *(T-C)	0.018	**0.008**	0.42	0.94	0.82	0.37	0.44	0.77

Haplotypes were estimated by using PHASE software (Stephens et al.). The association analyses and regression analyses for haplotypes were done by using Haplo. stats (Schaid et al.). *P *values for phenotypes were calculated from multiple linear regression analyses controlling for age, BMI, and number of parities as covariates except BMI phenotypes, which were adjusted for age and number of parities as covariates only. *P *values for phenotypes shown are co-dominant model. * *P *values for AUC_G, HOMA_B, HOMA_IR, and FPI were calculated after normalizing their data by applying logarithms

**Table 4:** MTNR1B haplotypes' genotype distribution, means and standard deviations of various phenotypes for GDM

Phenotype	Loci	C/C	C/R	R/R
BMI	*ht1*	518(22.34 ± 3.70)	904(22.34 ± 3.62)	454(22.35 ± 3.59)
	*ht2*	584(22.39 ± 3.64)	896(22.35 ± 3.63)	396(22.28 ± 3.62)
	*ht3*	1719(22.31 ± 3.58)	152(22.72 ± 4.04)	5(23.54 ± 6.94)
	*ht4*	1788(22.36 ± 3.65)	86(22.16 ± 3.21)	2(19.80 ± 1.41)
AUC_G	*ht1*	449(438.33 ± 74.35)	757(429.75 ± 79.48)	375(420.39 ± 84.28)
	*ht2*	480(420.98 ± 83.23)	753(430.50 ± 78.91)	348(441.20 ± 73.83)
	*ht3*	1446(430.46 ± 80.09)	130(425.51 ± 72.41)	5(405.10 ± 75.33)
	*ht4*	1516(429.95 ± 79.07)	64(431.35 ± 88.89)	1(361.50)
HOMA_B	*ht1*	509(225.47 ± 126.01)	898(240.16 ± 144.09)	448(254.96 ± 200.10)
	*ht2*	577(251.20 ± 186.38)	889(240.64 ± 143.02)	389(220.51 ± 128.44)
	*ht3*	1700(239.45 ± 157.09)	150(242.64 ± 138.61)	5(239.14 ± 23.78)
	*ht4*	1768(239.99 ± 157.73)	85(232.89 ± 98.82)	2(280.10 ± 66.75)
HOMA_IR	*ht1*	512(2.66 ± 1.60)	898(2.58 ± 1.45)	450(2.54 ± 1.50)
	*ht2*	579(2.54 ± 1.61)	889(2.59 ± 1.34)	392(2.68 ± 1.68)
	*ht3*	1705(2.59 ± 1.46)	150(2.65 ± 1.95)	5(2.58 ± 1.28)
	*ht4*	1773(2.60 ± 1.52)	85(2.42 ± 1.16)	2(2.00 ± 0.24)
FPI	*ht1*	512(12.34 ± 6.48)	898(12.09 ± 5.64)	450(12.00 ± 5.71)
	*ht2*	579(11.98 ± 6.12)	889(12.15 ± 5.31)	392(12.35 ± 6.78)
	*ht3*	1705(12.11 ± 5.74)	150(12.45 ± 7.54)	5(12.36 ± 4.49)
	*ht4*	1773(12.17 ± 5.96)	85(11.51 ± 4.61)	2(10.50 ± 0.71)
FPG	*ht1*	449(86.46 ± 12.66)	757(85.32 ± 11.89)	375(84.49 ± 13.30)
	*ht2*	480(84.49 ± 12.83)	753(85.40 ± 11.82)	348(86.86 ± 13.23)
	*ht3*	1446(85.53 ± 12.66)	130(84.65 ± 10.31)	5(82.20 ± 9.36)
	*ht4*	1516(85.44 ± 12.50)	64(85.64 ± 11.87)	1(80.00)

C/C, C/R, and R/R indicate major homozygote, heterozygote, and minor homozygote, respectively.

FPI, fasting plasma insulin; FPG, fasting plasma glucose

We have also performed regression analysis with various diabetes-related phenotypes, including body mass index (BMI, analyzed controlling for age and the number of parities as covariate), AUC-G, HOMA-B, HOMA-IR, FPI and FPG, with age, BMI, and numbers of parities as covariates (Table 5). From the analyses, both SNPs showed significant associations for AUC-G, HOMA-B, and FPG with phenotypes among all subjects (Table 5). The rare alleles of the 2 SNPs were associated with bigger AUC-G, lower HOMA-B, and higher FPG, and these alleles were the risk alleles for GDM (T for *rs1387153 *and G for *rs10830963*). To see if a certain SNP was functional, we have performed conditional analyses for GDM and other phenotypes. The results are listed in Table 6. From the results, *rs10830963 *retained its signal, while the significance of *rs1387153 *was disappeared, which suggest that *rs1387153 *might have shown its signal because of its LD with *rs10830963*, although not clear in GDM-related phenotype analyses.

**Table 5:** Multiple regression analyses of *MTNR1B *polymorphisms with diabetes-related phenotypes among all subjects

Phenotype	Loci	C/C	C/R	R/R	***Pacor***.	***Pbcor***.	***Pccor***.
BMI	*rs1387153*	548(22.38 ± 3.65)	888(22.37 ± 3.65)	445(22.24 ± 3.56)	1	1	1
	*rs10830963*	511(22.34 ± 3.60)	904(22.33 ± 3.58)	459(22.37 ± 3.75)	1	1	1
AUC_G*	*rs1387153*	454(419.91 ± 81.85)	743(431.31 ± 79.96)	386(439.26 ± 74.28)	**0.0007**	**0.004**	**0.03**
	*rs10830963*	419(421.23 ± 85.41)	763(429.09 ± 78.39)	403(439.14 ± 74.58)	**0.002**	**0.04**	**0.03**
HOMA_B*	*rs1387153*	541(252.38 ± 190.86)	882(239.90 ± 143.11)	437(222.90 ± 125.81)	**0.01**	0.29	0.07
	*rs10830963*	505(251.36 ± 191.00)	897(240.78 ± 143.84)	451(223.74 ± 128.37)	**0.03**	0.72	0.04
HOMA_IR*	*rs1387153*	543(2.53 ± 1.63)	882(2.59 ± 1.35)	440(2.64 ± 1.62)	1	1	1
	*rs10830963*	507(2.53 ± 1.47)	897(2.57 ± 1.43)	454(2.69 ± 1.65)	1	1	1
FPI*	*rs1387153*	543(11.98 ± 6.21)	882(12.15 ± 5.34)	440(12.26 ± 6.56)	1	1	1
	*rs10830963*	507(11.91 ± 5.59)	897(12.07 ± 5.61)	454(12.44 ± 6.64)	1	1	1
FPG	*rs1387153*	454(84.30 ± 12.71)	743(85.55 ± 12.01)	386(86.58 ± 12.94)	**0.01**	0.07	0.22
	*rs10830963*	419(84.63 ± 13.24)	763(85.13 ± 11.62)	403(86.71 ± 12.99)	**0.04**	0.72	0.07

*P *value was calculated from multiple linear regression analyses controlling for age, BMI, and number of parities as covariates except BMI phenotypes, which was adjusted for age and number of parities as covariates only. *P*a, *P*b, and *P*c designate *P *values for co-dominant, dominant, and recessive models, respectively. *P *values were corrected for multiple testing by multiplying the number of tests (36 tests, 2 SNPs × 3 models × 6 phenotypes) (Bonferroni correction). C/C, C/R, and R/R indicate major homozygote, heterozygote, and minor homozygote, respectively. * *P *values for AUC_G, HOMA_B, HOMA_IR, and FPI were calculated after normalizing their data by applying logarithms (Data shown are raw values). FPI, fasting plasma insulin; FPG, fasting plasma glucose

**Table 6:** Conditional association analyses of *MTNR1B *genetic variants

Phenotype	Loci	P	Conditioned P value by	
			
			*rs1387153*	*rs10830963*
GDM	*rs1387153*	0.00008	-	**0.007**
	*rs10830963*	0.00001	0.22	-
				
BMI	*rs1387153*	0.66	-	0.59
	*rs10830963*	0.77	0.51	-
				
AUC_G*	*rs1387153*	0.00002	-	0.34
	*rs10830963*	0.00006	0.11	-
				
HOMA_B*	*rs1387153*	0.0004	-	0.25
	*rs10830963*	0.0007	0.07	-
				
HOMA_IR*	*rs1387153*	0.15	-	0.28
	*rs10830963*	0.14	0.12	-
				
FPI*	*rs1387153*	0.58	-	0.89
	*rs10830963*	0.49	0.49	-
				
FPG	*rs1387153*	0.0004	-	0.36
	*rs10830963*	0.001	0.85	-

Conditional P values were estimated and permutated using the software PLINK (Purcell et al.). * *P *values for AUC_G, HOMA_B, HOMA_IR, and FPI were calculated after normalizing their data by applying logarithms

## Discussion

In previous studies on the *rs1387153 *and *rs10830963*, researchers found strong associations between the two SNPs of *MTNR1B *and T2DM, and also with FPG levels, which is an important phenotype for diabetes. A study in European population, which included French and Danish among other nations, showed that *rs1387153 *was significantly associated with FPG level (*P *= 1.3 × 10^-7^, adjusted genome-wide *P *= 0.04) and T2DM (OR (95% CI) = 1.15(1.08-1.22), *P *= 6.3 × 10^-5^). Another study in European population found significant associations of *rs10830963 *with FPG (*P *= 3.2 × 10^-50^) and T2DM (OR (95% CI) = 1.09(1.05-1.12), *P *= 3.3 × 10^-7^). The Two SNPs were closely related with each other, as evidenced by the linkage disequilibrium test (|D'| = 0.89) in our study. Their haplotype analyses results showed that *ht1 *(C/C) and *ht2 *(T/G) were mostly tagged by *rs10830963 *and *rs1387153*, respectively (>92%). Therefore, *ht1/ht2 *showed similar associations with each SNP, respectively.

Here, we performed the association studies in Korean pregnant women and we found significant associations between the SNPs and GDM, with enough samples for high statistical power. It is well known that T2DM and GDM are closely related diseases, since they exhibit similar characteristics such as glucose intolerance. However, there had yet to be a study that looked into the association between the polymorphisms of *MTNR1B *and GDM, and our study confirmed the relations between the two. Also, we carried out regression analyses between the polymorphisms of *MTNR1B *and various phenotypes including FPG. Although both SNPs showed associations with GDM and FPG, our results suggested that the two genetic variants of *MTNR1B *were stronger risk factors for GDM in Korean population compared to the previous results for T2DM in European population (OR (95% CI) = 1.44 (1.17-1.78) for *rs1387153 *in dominant inheritance model and 1.46 (1.18 - 1.81) for *rs10830963 *in the present study and OR = 1.15 (1.08-1.22) for *rs1387153 *and OR = 1.09 (1.05-1.12) for *rs10830963 *in the two previous studies). We suspect that the genetic differences between GDM and T2DM and the population difference between Europeans and Asians could have contributed to this result. Previously, there have been a few cases where a gene associated with T2DM was not associated with GDM at all [20], or showed different effect sizes [21,22], and our results suggest that *MTNR1B *affects T2DM and GDM in varying degrees as well.

Moreover, our results suggest that the two polymorphisms investigated are associated with beta-cell function (Table 5). Association between beta-cell function and *MTNR1B *was previously reported [18,23], which shows that we were able to replicate the result in Korean population, strengthening the notion that *MTNR1B *polymorphisms are related with impaired beta-cell function. Recently, several groups of scientists have studied the association between the gene variants of *MTNR1B *and glucose tolerances. *rs10830963 *was found to be associated with FPG and decreased beta-cell function in a group of obese children, which is consistent with our finding [24]. Three independent studies of the *MTNR1B *genetic variants in Han Chinese subjects also found significant associations for increased FPG, impaired beta-cell function, glycated hemoglobin, and T2DM [18,25,26]. Furthermore, a study with European populations also found significant associations between variations of *MTNR1B *with BMI and FPG, but not with maturity-onset diabetes of the young (MODY) or T2DM [27]. Although some of their results do not agree with each other in the association with T2DM, these findings firmly back up the association between the SNPs in our study and FPG or impaired beta-cell function. On the other hand, we could not find any significant associations between insulin resistance (HOMA-IR) and the two genetic variants. Since our results are backed with high statistical power, this leads us to conclude that two genetic variants of *MTNR1B *may be associated with the disease by affecting glucose metabolism through impaired insulin secretion, as previously suggested by other studies with *KCNQ1 *genetic variants [8,18,28].

In addition, further conditional analyses results suggested that *rs10830963 *might be more likely functional in case/control analysis, although not clear in GDM-related phenotype analyses.

Although our results showed the significant associations with GDM and several diabetic characteristics, there are a couple of limitations. First, our study only concentrated on pregnant women among Korean population, so we cannot conclude that the *MTNR1B *gene variants are associated with FPG or impaired beta-cell function in all Korean population. Also, even though our study strongly suggests that the SNPs may also be associated with T2DM in Korean population, this is not confirmed yet. Any further studies on these two genetic variants in Korean population should concentrate on these parts.

## Conclusions

The present study showed that two *MTNR1B *polymorphisms were associated with increased risk for GDM in Korean female population. Two polymorphisms *rs1387153 *and *rs10830963 *also showed significant associations with FPG and beta-cell function, but not with insulin resistance. Further conditional analyses results suggested that *rs10830963 *might be more likely functional in case/control analysis, although not clear in GDM-related phenotype analyses. The effective sizes found between the two polymorphisms and FPG was stronger compared to previous studies, which is possibly due to the genetic difference between European and Korean populations, or the difference between GDM and T2DM. Based on the current results, we suspect that these two polymorphisms will have significant associations with increased risk of GDM in other populations as well. Also, our discovery would be helpful for understanding of genetic etiology of GDM as well.

## Competing interests

The authors declare that they have no competing interests.

## Authors' contributions

JYK and HSC developed tables/figures, and wrote the manuscript. SHB, SWL, SMP, MHK, JHC, JSC, MYK, JHY, and DHC helped recruiting subjects, conducted experiments, and collected data. HSC and BLP analyzed data by performing statistical analysis. HDS and SHK managed all of the study and helped to draft the manuscript. All authors have read and approved the final manuscript.

## Pre-publication history

The pre-publication history for this paper can be accessed here:

http://www.biomedcentral.com/1471-2350/12/82/prepub

## References

[B1] Cho YM Kim TH Lim S Choi SH Shin HD Lee HK Park KS Jang HC Type 2 diabetes-associated genetic variants discovered in the recent genome-wide association studies are related to gestational diabetes mellitus in the Korean population Diabetologia 2009 12 253 261 10.1007/s00125-008-1196-4 19002430

[B2] Metzger BE Summary and recommendations of the Third International Workshop-Conference on Gestational Diabetes Mellitus Diabetes 1991 12 Suppl 2 197 201 1748259 10.2337/diab.40.2.s197

[B3] Amankwah K Prentice R Fleury F The incidence of gestational diabetes Obstet Gynecol 1977 12 497 498 857215

[B4] Jovanovic L Pettitt DJ Gestational diabetes mellitus Jama 2001 12 2516 2518 10.1001/jama.286.20.2516 11722247

[B5] Buchanan TA Xiang AH Gestational diabetes mellitus J Clin Invest 2005 12 485 491 15765129 10.1172/JCI24531 PMC1052018

[B6] Evans JC Frayling TM Cassell PG Saker PJ Hitman GA Walker M Levy JC O'Rahilly S Rao PV Bennett AJ Jones EC Menzel S Prestwich P Simecek N Wishart M Dhillon R Fletcher C Millward A Demaine A Wilkin T Horikawa Y Cox NJ Bell GI Ellard S McCarthy MI Hattersley AT Studies of association between the gene for calpain-10 and type 2 diabetes mellitus in the United Kingdom Am J Hum Genet 2001 12 544 552 10.1086/323315 11481585 PMC1235484

[B7] Salonen JT Uimari P Aalto JM Pirskanen M Kaikkonen J Todorova B Hypponen J Korhonen VP Asikainen J Devine C Tuomainen TP Luedemann J Nauck M Kerner W Stephens RH New JP Ollier WE Gibson JM Payton A Horan MA Pendleton N Mahoney W Meyre D Delplanque J Froguel P Luzzatto O Yakir B Darvasi A Type 2 diabetes whole-genome association study in four populations: the DiaGen consortium Am J Hum Genet 2007 12 338 345 10.1086/520599 17668382 PMC1950819

[B8] Prokopenko I Langenberg C Florez JC Saxena R Soranzo N Thorleifsson G Loos RJ Manning AK Jackson AU Aulchenko Y Variants in MTNR1B influence fasting glucose levels Nat Genet 2009 12 77 81 10.1038/ng.290 19060907 PMC2682768

[B9] Bouatia-Naji N Bonnefond A Cavalcanti-Proenca C Sparso T Holmkvist J Marchand M Delplanque J Lobbens S Rocheleau G Durand E A variant near MTNR1B is associated with increased fasting plasma glucose levels and type 2 diabetes risk Nat Genet 2009 12 89 94 10.1038/ng.277 19060909

[B10] Peschke E Frese T Chankiewitz E Peschke D Preiss U Schneyer U Spessert R Muhlbauer E Diabetic Goto Kakizaki rats as well as type 2 diabetic patients show a decreased diurnal serum melatonin level and an increased pancreatic melatonin-receptor status J Pineal Res 2006 12 135 143 10.1111/j.1600-079X.2005.00287.x 16441550

[B11] Lee YH Kang ES Kim SH Han SJ Kim CH Kim HJ Ahn CW Cha BS Nam M Nam CM Lee HC Association between polymorphisms in SLC30A8, HHEX, CDKN2A/B, IGF2BP2, FTO, WFS1, CDKAL1, KCNQ1 and type 2 diabetes in the Korean population J Hum Genet 2008 12 991 998 10.1007/s10038-008-0341-8 18991055

[B12] Carpenter MW Coustan DR Criteria for screening tests for gestational diabetes Am J Obstet Gynecol 1982 12 768 773 7148898 10.1016/0002-9378(82)90349-0

[B13] Matthews DR Hosker JP Rudenski AS Naylor BA Treacher DF Turner RC Homeostasis model assessment: insulin resistance and beta-cell function from fasting plasma glucose and insulin concentrations in man Diabetologia 1985 12 412 419 10.1007/BF00280883 3899825

[B14] Krzyzanowska K Zemany L Krugluger W Schernthaner GH Mittermayer F Schnack C Rahman R Brix J Kahn BB Schernthaner G Serum concentrations of retinol-binding protein 4 in women with and without gestational diabetes Diabetologia 2008 12 1115 1122 10.1007/s00125-008-1009-9 18437353 PMC2676863

[B15] Livak KJ Allelic discrimination using fluorogenic probes and the 5' nuclease assay Genet Anal 1999 12 143 149 10084106 10.1016/s1050-3862(98)00019-9

[B16] Barrett JC Fry B Maller J Daly MJ Haploview: analysis and visualization of LD and haplotype maps Bioinformatics 2005 12 263 265 10.1093/bioinformatics/bth457 15297300

[B17] Menashe I Rosenberg PS Chen BE PGA: power calculator for case-control genetic association analyses BMC Genet 2008 12 36 18477402 10.1186/1471-2156-9-36 PMC2387159

[B18] Zhou Q Zhang K Li W Liu JT Hong J Qin SW Ping F Sun ML Nie M Association of KCNQ1 gene polymorphism with gestational diabetes mellitus in a Chinese population Diabetologia 2009 12 2466 2468 10.1007/s00125-009-1500-y 19714318

[B19] Stephens M Smith NJ Donnelly P A new statistical method for haplotype reconstruction from population data Am J Hum Genet 2001 12 978 989 10.1086/319501 11254454 PMC1275651

[B20] Shaat N Lernmark A Karlsson E Ivarsson S Parikh H Berntorp K Groop L A variant in the transcription factor 7-like 2 (TCF7L2) gene is associated with an increased risk of gestational diabetes mellitus Diabetologia 2007 12 972 979 10.1007/s00125-007-0623-2 17342473

[B21] Grant SF Thorleifsson G Reynisdottir I Benediktsson R Manolescu A Sainz J Helgason A Stefansson H Emilsson V Helgadottir A Styrkarsdottir U Magnusson KP Walters GB Palsdottir E Jonsdottir T Gudmundsdottir T Gylfason A Saemundsdottir J Wilensky RL Reilly MP Rader DJ Bagger Y Christiansen C Gudnason V Sigurdsson G Thorsteinsdottir U Gulcher JR Kong A Stefansson K Variant of transcription factor 7-like 2 (TCF7L2) gene confers risk of type 2 diabetes Nat Genet 2006 12 320 323 10.1038/ng1732 16415884

[B22] Leipold H Knoefler M Gruber C Huber A Haslinger P Worda C Peroxisome proliferator-activated receptor gamma coactivator-1alpha gene variations are not associated with gestational diabetes mellitus J Soc Gynecol Investig 2006 12 104 107 10.1016/j.jsgi.2005.12.004 16443502

[B23] Staiger H Machicao F Schafer SA Kirchhoff K Kantartzis K Guthoff M Silbernagel G Stefan N Haring HU Fritsche A Polymorphisms within the novel type 2 diabetes risk locus MTNR1B determine beta-cell function PLoS One 2008 12 e3962 10.1371/journal.pone.0003962 19088850 PMC2597741

[B24] Holzapfel C Siegrist M Rank M Langhof H Grallert H Baumert J Irimie C Klopp N Wolfarth B Illig T Hauner H Halle M Association of a MTNR1B gene variant with fasting glucose and HOMA-B in children and adolescents with high BMI-SDS Eur J Endocrinol 2010 10.1530/EJE-10-0588 21059861

[B25] Kan MY Zhou DZ Zhang D Zhang Z Chen Z Yang YF Guo XZ Xu H He L Liu Y Two susceptible diabetogenic variants near/in MTNR1B are associated with fasting plasma glucose in a Han Chinese cohort Diabet Med 2010 12 598 602 10.1111/j.1464-5491.2010.02975.x 20536959

[B26] Tam CH Ho JS Wang Y Lee HM Lam VK Germer S Martin M So WY Ma RC Chan JC Ng MC Common polymorphisms in MTNR1B, G6PC2 and GCK are associated with increased fasting plasma glucose and impaired beta-cell function in Chinese subjects PLoS One 2010 12 e11428 10.1371/journal.pone.0011428 20628598 PMC2900202

[B27] Andersson EA Holst B Sparso T Grarup N Banasik K Holmkvist J Jorgensen T Borch-Johnsen K Egerod KL Lauritzen T Sorensen TI Bonnefond A Meyre D Froguel P Schwartz TW Pedersen O Hansen T The MTNR1B G24E variant associates with BMI and fasting plasma glucose in the general population in studies of 22,142 Europeans Diabetes 2010 10.2337/db09-1757 PMC2874716 20200315

[B28] Mussig K Staiger H Machicao F Kirchhoff K Guthoff M Schafer SA Kantartzis K Silbernagel G Stefan N Holst JJ Gallwitz B Haring HU Fritsche A Association of type 2 diabetes candidate polymorphisms in KCNQ1 with incretin and insulin secretion Diabetes 2009 12 1715 1720 10.2337/db08-1589 19366866 PMC2699873

